# Advancements in the Field of Protein-Based Hydrogels: Main Types, Characteristics, and Their Applications

**DOI:** 10.3390/gels11050306

**Published:** 2025-04-22

**Authors:** Gábor Katona, Bence Sipos, Ildikó Csóka

**Affiliations:** Institute of Pharmaceutical Technology and Regulatory Affairs, University of Szeged, Eötvös Street 6., H-6720 Szeged, Hungary; katona.gabor@szte.hu (G.K.); csoka.ildiko@szte.hu (I.C.)

**Keywords:** protein, hydrogel, collagen, serum albumin, gelatin, silk fibroin, elastin, fibrin, regenerative medicine

## Abstract

Regenerative medicine is a challenging field in current research and development, whilst translating the findings of novel tissue regenerative agents into clinical application. Protein-based hydrogels are derived from various sources, with animal-derived products being primarily utilized to deliver cells and promote cell genesis and proliferation, thereby aiding in numerous indications, including bone tissue regeneration, cartilage regeneration, spinal cord injury, and wound healing. As biocompatible and biodegradable systems, they are tolerated by the human body, allowing them to exert their beneficial effects in many indications. In this review article, multiple types of animal-derived proteins (e.g., collagen, gelatin, serum albumin, fibrin) were described, and a selection of the recent literature was collected to support the claims behind these innovative systems. During the literature review, special indications were found when applying these hydrogels, including the therapeutic option to treat post-myocardial infarct sites, glaucoma, and others. Maintaining their structure and mechanical integrity is still challenging. It is usually solved by adding (semi)synthetic polymers or small molecules to strengthen or loosen the mechanical stress in the hydrogel’s structure. All in all, this review points out the potential application of value-added delivery systems in regenerative medicine.

## 1. Introduction

Chemically synthesized excipients play a crucial role in current research and development processes, aiding the more efficient utilization of novel drugs or carrier systems. Hydrogels are of paramount importance, since they can be used for the sustained release of drugs whilst meeting the therapeutic requirements of various active substances [1,2,3]. These polymers, lipids, or inorganic particles, capable of forming hydrogels, are generally biocompatible and biodegradable. Still, they are surpassed in their biomimetic nature by natural-origin or derived proteins, which are also suitable for drug or cell delivery [4,5,6]. Numerous proteins can form gels independently due to their biological function. Still, many can be physically or chemically engineered to form stable polymeric matrices, controlling drug release or acting as natural tissue fillers. These proteins have excellent biocompatibility and biodegradability and exert no (or minimal) immunogenic effect on the human body. They degrade into non-harmful byproducts with reduced adverse effects whilst eliminating the need for surgical removal [7,8,9,10].

They are versatile in their range of applications. The drug delivery system can encapsulate different therapeutic agents, such as small-molecule drugs, other proteins or peptides, nucleic acids like DNA and RNA, and live cells, including stem cells. Generally, the most common application routes include topical administration or on mucosal surfaces, such as nasal or ocular administration routes [11,12,13]. Intelligent, stimuli-sensitive systems can also be engineered, which are usually administered intravenously or subcutaneously to target a specific site in the body, typically for deeper tissues, such as bones, cartilage, or tumor cells. Their general application areas include cutting-edge fields, such as tissue engineering, wound healing, 3D bioprinting, vaccine delivery, and cancer therapy. Regenerative medicine also plays a crucial role in the utilization of these polymers. Acute clinical applications are also considered in this area; not only can the typical dosage forms be exploited, but plasters, scaffolds, or implants can be utilized to exert their beneficial effects [14,15,16,17,18,19,20]. The main advantages and disadvantages (translated into technological challenges) can be seen in Table 1.

**Figure 1:**
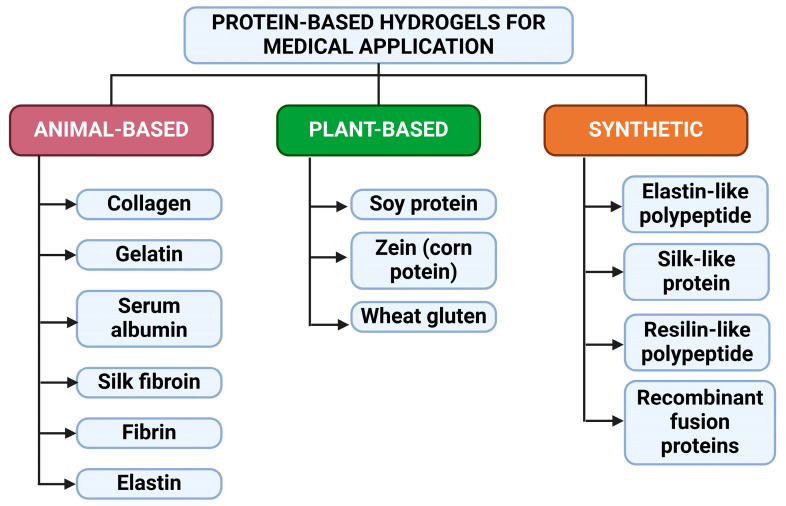
Leading groups of protein-based hydrogels for medical applications. Created with biorender.com with permission.

Multiple types of protein can be applied for making hydrogels, generally, due to their water solubility. Thus, it is technologically difficult to form oleogels. Based on their origin, there are three main classes of protein (Figure 1). Natural animal-derived proteins include biocompatible and biodegradable proteins, mainly from bovine, porcine, or fish origin, such as collagen, gelatin, or elastin, but other animal products can also be found, such as silk fibroin produced by silkworms [21,22]. Despite their natural origins and biocompatibility, proper extraction and manufacturing techniques are paramount, since additional byproducts or contaminants may be contained in these products. This can lead to immunogenic responses and inflammation if not treated properly. Plant-derived proteins are also applicable as they offer sustainability and ethical alternatives to animal proteins, and since some religions exclude using bovine or porcine-derived proteins. Common plant-derived proteins include zein, soy protein, and wheat gluten, but they are generally used for other dosage forms to strengthen the hydrogels. The most common application of these proteins is in the food industry. With the constant evolution of biotechnology, recombinant and (semi)synthetic proteins can be produced via genetic engineering. This can help to increase the stability of the hydrogels in the human body, since they are susceptible to quick degradation in the body. If the gel structure is not firm enough, it can be quickly degraded via fast metabolism. Thus, application of crosslinkers is advised to enhance stability, and genetic engineering helps tackle this challenge. This includes engineering mostly animal-based proteins, such as elastin-like recombinamers, recombinant collagen or silk proteins, and other peptide-based, self-assembling hydrogels [21,23,24,25,26].

Regulatory concerns also affect protein-based hydrogels generally. They can be categorized into multiple medical groups, from drugs to combined medical devices. Typically, biologics are one of the complex drug delivery systems, if an active pharmacological effect is expected among them. However, they can act alone, supporting tissue regeneration without pharmacological effects, stimulating the microenvironment in the damaged area, making it possible to call them medical devices. They can also be utilized for carrier systems for drugs or cells, making them a drug + device combined product, making the authorization process harder, as the clinical trials should fit the criteria of the Clinical Trials Directive for drugs and the Medical Device Regulation in the European Union [27,28].

This review article focused on the main types and relevance of the animal-derived proteins and their applicability to medicine. Regenerative medicine, including wound healing and bone or cartilage regeneration, is one of the main applications of this type of hydrogel. However, some of them can be utilized for applications in drug or cell delivery. This is especially useful to enhance tissue regeneration further, as the delivery of progenitor cells through conventional administration is insufficient or requires more prolonged, more frequent administration.

## 2. Collagen-Based Hydrogels for Medicinal Applications

### 2.1. General Structure, Gelling Mechanism, and Properties of Collagen

**Figure 2:**
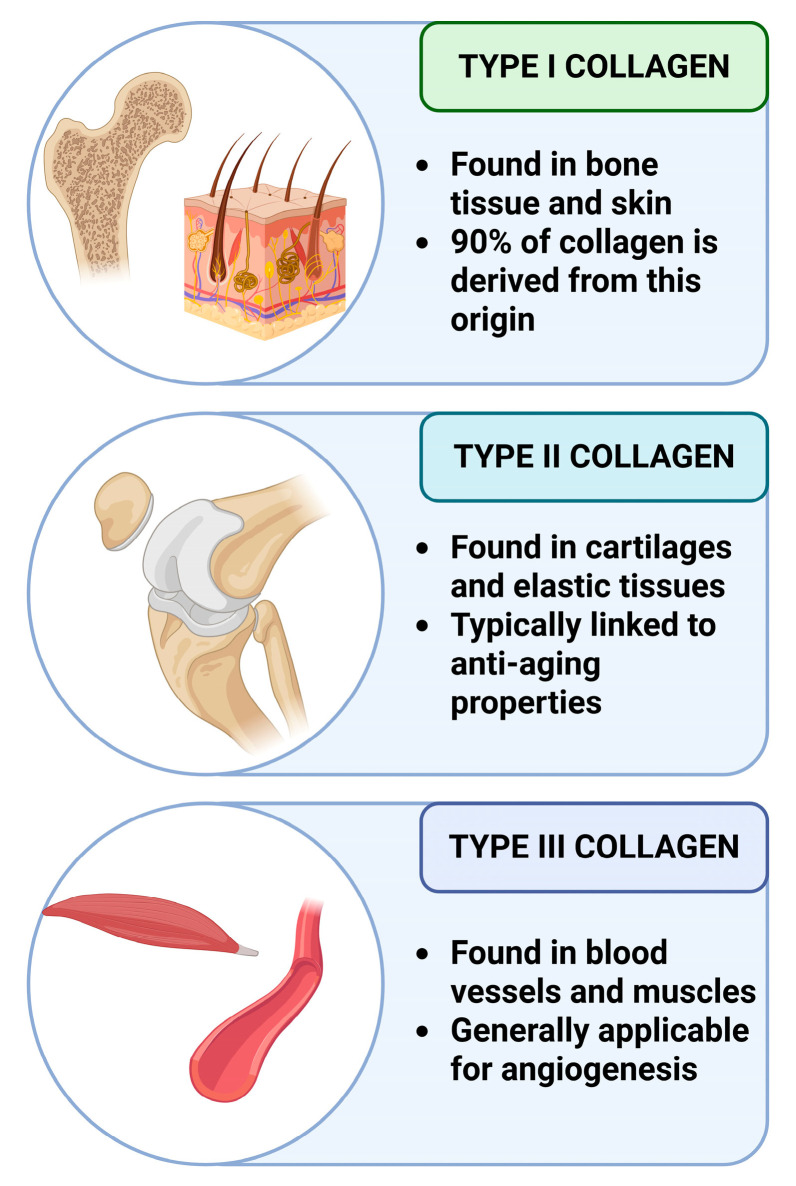
The main types of collagen used in medicine originate in mammal bodies. Created with biorender.com with permission.

Collagen is a structural protein that can be found in the extracellular matrix of animals. It maintains the strength and integrity of several tissues, especially in the skin, bones, cartilage, and tendons (Figure 2). There are multiple types of collagen based on their origin and structural differences. Type I collagen is the most widely used drug delivery system since it is the most abundant and easiest to extract, capable of forming strong fibrils, and is ideal for gel formation. It is bovine, porcine, or fish-originated and can be applied for wound healing, bone regeneration, or to form injectable hydrogels. Type II collagen is utilized for its targetability, specifically for cartilage-targeted delivery, since it can also be found at high concentrations in human cartilage. Type III is worth mentioning; however, it is usually combined with Type I collagen for skin regeneration and wound healing [29,30,31].

Collagen has numerous advantages for utilization as a drug delivery system. Since it is a natural protein found abundantly in the human body, it is well-tolerated by the immune system, and is thus biocompatible and biodegradable. From the technological aspects of utilization of collagen, numerous dosage forms can be formulated, including hydrogels, micro- or nanospheres, and scaffolds. The formed matrix can encapsulate drugs, and, at the gel-forming concentrations, a sustained release can usually be achieved, which can be influenced by crosslinking with polymers. Numerous sites can be found in the collagen’s structure to offer chemical modification, which is most auspicious for attachment of ligands to achieve targeted drug delivery [29,32]. Despite all these advantages, some disadvantages are also found in the case of collagen. Since it is derived from animal sources, it is hard to obtain a consistent batch each time due to the variety in purity, composition, and mechanical properties such as tensile strength, etc. Bovine- and porcine-derived materials are also prone to the risk of immunogenicity and contamination if not processed correctly. Collagen is rarely used alone, since, without modifications, it can degrade rapidly in the human body, hindering its controlled release profile. Highly hydrophobic compounds or active substances with large molecular weights are also challenging to encapsulate in the collagen matrices due to steric hindrance and high interactions that repel the drug from its matrix. From the industrial point of view, it is still a costly process to produce pharmaceutical-grade collagen, which hinders large-scale production. Due to the variety in the chemical structure based on its origin, standardization is challenging [33,34,35,36].

Gel formation of collagen is favorable; however, it is a multistep process that needs careful formulation strategies. First, collagen must be in a soluble form, where the monomeric collagen is often dissolved in neutral buffered solutions or slightly acidic conditions at low temperatures. In this form, it is typically a triple-helical protein, and this triple helix is stabilized by hydrophobic interactions or hydrogen bonds amongst the α-chains. The next step is to initiate fibrillogenesis, which is the self-assembly of the collagen monomers at the physiological temperature range at neutral pH. It is a spontaneous process where non-covalent interactions guide the alignment of monomers. During the lag phase, small oligomers form from the monomers, which act as nuclei to catalyze fibril formation. This is followed by the constant buildup by elongation of monomers on the nucleated fibrils in a quarter-staggered arrangement. After the collagen fibrils become entangled to a density and thickness dependent on the concentration, this percolating 3D network traps water inside and forms viscoelastic hydrogels. Due to its complexity, strong mechanical strength can be achieved alongside proper consistency and spreadability, which is required by semi-solid dosage forms. Crosslinking is also advisable to ensure increased mechanical strength at the final step. Various materials have been utilized for this, such as glutaraldehyde, genipin, and EDC [37,38,39,40,41].

### 2.2. Recent Advancements in Collagen-Based Drug/Cell-Delivery Systems

Based on its anatomical and physiological function, the main areas of application of collagen include bone tissue and cartilage regeneration. Since collagen is also found in skin tissue, wound healing and skin tissue regeneration are also paramount for such hydrogels. Moeinzadeh et al. developed a novel formulation where sodium alginate and collagen hydrogels were combined to evaluate the effect of shear-thinning hydrogels, providing a stable matrix for cell and growth factor delivery. The optimized formulation successfully acted as an injectable formulation through 20 G needles, which remained stable for up to 28 days of storage, supporting long-term usability. The porous microstructure of the combined hydrogel had an average pore size of 15 µm. The formulation had a sustained release profile; whilst under osteogenic conditions, the activity of ALP and calcium content increased significantly, which confirms osteogenic differentiation. In vivo rat calvarial bone defect model studies also proved that the growth factor-loaded combined hydrogel increased bone volume by almost double during the investigation period [32]. Hwang et al. also developed a dual-functional alginate and collagen-based hydrogel, which may be an efficient drug delivery system for cancer treatment. The thermally responsive hydrogel was incorporated with the immune stimulator polyionosinic:polycytidylic acid and indocyanine green, and an evaluation was performed to map its anticancer and anti-metastatic effects on CT-26 lung carcinoma and 4T1 breast tumor. Therapeutic efficiency was proven in combination with irradiation of photothermal therapy. The results demonstrated that the efficient drug delivery system loaded with the immune stimulator active substance prevented lung metastasis and eliminated the original breast tumor as well [42]. Zhao et al. also prepared injectable hydrogels crosslinked with microbial transglutaminase, which is a promising crosslinker since the excess can be eliminated more easily from the hydrogel matrices compared to other, potentially toxic and non-biocompatible crosslinkers. In their study, an injectable sol-state hydrogel was administered locally, followed by in situ gelling. Incorporating human-like collagen (HLC) demonstrated high cell viability upon administration, and the mechanical properties were appropriate to aid wound healing due to adequate elasticity and strength [43].

Gao et al. formulated self-crosslinkable hydrogels based on collagen for efficient cell encapsulation. Their study utilized collagen type I with activated chondroitin sulfate, which acts as a self-crosslinker without requiring additive catalysts. Chondrocytes were encapsulated in the hydrogel matrix, and the results suggest that the hydrogel supported the survival and extracellular matrix secretion of chondrocytes, supporting tissue regeneration and engineering. The mechanical properties of the hydrogels also supported injectability and proper strength, further supporting their applicability for cell delivery to promote cell proliferation [44]. Gilarska et al. tested various hydrogels based on collagen in combination with hyaluronic acid and chitosan. Via the optimization of hyaluronic acid and the concentration of the crosslinker genipin, tunable, biomimetic hydrogels were formulated that were suitable for injection. Their biocompatible nature allows these hydrogels to form injectable scaffolds, which is paramount for tissue engineering. Bone regeneration is a challenging medical field, especially for small bone losses. Via the utilization of this combined hydrogel, proliferation of new bone material was achieved without showing a significant cytotoxic effect [45]. Hyaluronic acid also demonstrated promising effects in the studies by Liu and Fan. A combined hydrogel capable of injectability to form scaffolds based on hyaluronic acid-tyrosine and human-like collagen was developed. As a crosslinker, 1,4-butanedioldiglycidyl ether (BDDE) was used. The physicochemical properties of the hydrogels are sufficient for them to act as soft tissue fillers due to their favorable swelling ratio, strength, and thermal stability in human conditions. Crosslinked hydrogels also proved to degrade more slowly than non-crosslinked ones, in a biocompatible manner. The biocompatibility was also tested via hematoxylin–eosin staining and immunohistochemical investigations, where the injected hydrogels caused a less intense inflammatory response [46].

Bone tissue engineering is challenging, as the rigid architecture of implantable scaffolds offers less diversity and limited ability to fill the irregular contours of bone defects. Hydroxyapatite is generally applied in solid scaffolds, but it can also be incorporated into soft scaffolds, which Bendtsen and Wei have investigated. In their study, alginate and collagen were used as an injectable hydrogel stabilized by calcium and phosphate. Gelation time was adequate upon injection, followed by a uniform distribution across the bone tissue to ensure an efficient filler to enhance bone regeneration and host–implant integration [47]. Self-crosslinking is advantageous for biomimetic formulations as no additional excipients must be administered. Yao et al. developed a di-self-crosslinking hyaluronan-based hydrogel in combination with type I collagen. A rapid thiol/maleimide click chemistry reaction was performed, supported by a thiol oxidation reaction, to serve as the self-catalyst for forming a crosslinked network. Their results proved that great promotion of cartilaginous tissue formation was achieved in vivo, and the lack of adhesion sites resulted in the untenable situation of maintaining effective connections amongst newborn cell clusters. The improved resistance to degradation and the chondrocyte adhesion also proved that it might be a potential clinical treatment option for the construction of injectable cartilage repair fillers. The mechanical strength observed is also a cause of this phenomenon, and its bioactive therapeutic efficiency can remain for a more extended period [48]. Carbon dot nanoparticles can also be integrated into collagen-based gels, which can be crosslinked with genipin to form injectable hydrogels. Similar to the studies conducted by Hwang et al., photodynamic therapy was utilized in the studies by Lu et al. to facilitate the effect on cartilage repair [42,49]. As the crosslinked hydrogels were formulated, their stiffness increased significantly in the presence of carbon dot nanoparticles. This was aided by the photodynamic therapy-assisted production of reactive oxygen species (ROS). These factors improved chondrogenic differentiation of bone marrow-derived stem cells, aiding cartilage defect repair. The combination of photodynamic therapy with the administration of hydrogels increased stem cell proliferation by more than 50%, subsequently increasing this ratio up to 3 weeks. As a minimally invasive repair option, this approach may be used to treat cartilage defects [49]. Icariin is a natural flavonoid that stimulates bone formation via osteoblast proliferation and differentiation. Liu et al. functionalized collagen/hyaluronic acid hydrogels with thiolated icariin to promote cartilage formation. The administered thiolated icariin decreased the cytotoxicity of icariin by itself, but the thiolation also promoted encapsulation into the hydrogel matrices. A facilitated chondrocyte proliferation was evaluated whilst maintaining chondrocyte phenotype, and promoted secretion of the cartilage’s extracellular matrix [50]. In another study, Gilarska et al. tested antimicrobial structurally stable injectable hydrogels based on collagen, chitosan, and lysine, functionalized via hyaluronic acid. The main advantage of this delivery system included its intrinsic antibacterial activity against *Escherichia coli*. The high porosity of the hydrogels offered great injectability in a biocompatible manner. The injected hydrogels also supported proliferation and adhesion of osteoblast-like cells, followed by alkaline phosphatase expression [51]. Time is a crucial factor in wound repair. Zhang et al. developed a super-ductile formulation based on collagen and capable of being injected and providing fast self-healing properties. Multiple functionalization-aiding excipients were added to each formulation, including guar gum, poly(N-isopropylacrylamide) (PNIPAM), graphene oxide, and borax to form reversible or permanent networks. The injectable hydrogels stretched by large amounts without rupturing, up to 100 cm long. The hydrogels self-heal within less than 3 min, showing excellent thermal sensitivity and near-infrared-responsive properties. The administered collagen–guar gum and PNIPAM-based hydrogels exerted the highest healing ratio on mouse skin wounds compared to the other formulations [52]. In Table 2, a collective summary can be found regarding the utilization of collagen for medical applications.

## 3. Gelatin-Based Hydrogels for Medicinal Applications

### 3.1. General Structure, Gelling Mechanism, and Properties of Gelatin

Gelatin is widely applied in the food industry, but there are numerous advantages to its use in the pharmaceutical industry as well. It is a natural, biocompatible, and biodegradable protein, which is water-soluble. Gelatin is a polypeptide mixture of high glycine, proline, and hydroxyproline ratios. Its triple helix is denatured, and the chains become random coils compared to collagen. It is also characterized by a thermo-reversible gelling nature, which will melt upon heating and only set when it is cooled down. It is manufactured from collagen via hydrolysis in acidic or alkaline treatment after removing fats, non-collagenous proteins, and minerals from animals’ skin, bones, or connective tissues. During the preparation, hot water extraction is used to solubilize gelatin and it is filtered to separate it from other materials. The final gelatin is usually dried into powders or sheets. There are three main gelatine types: type A, from porcine origin; type B, from bovine origin; and the last is fish gelatin. The kind of gelatine also determines the production method: for porcine skin, acid hydrolysis is used as its isoelectric point is 9, providing faster gelation and lower viscosity, whilst, on the other hand, bovine hide and bones undergo alkaline hydrolysis with an isoelectric point of 5, ensuring slower gelation with higher viscosity [53,54,55].

**Figure 3:**
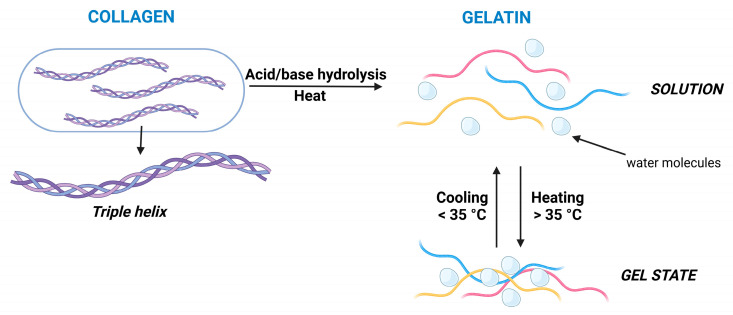
Schematics of the production of gelatin and its gelation mechanism via temperature. Created with biorender.com with permission.

The mechanism of gelation is opposite to that of collagen, in which collagen monomers are dissolved in cold water, followed by heating up to achieve elastic gels; in the case of gelatine, first, gelatin should be dissolved at higher temperatures (40–50 °C) resulting in a sol state, which solidifies into elastic gels upon cooling down [55,56,57]. During the cooling process, the polypeptide chains reassociate into partial triple helices, and the helix nuclei act as junction zones to expand the 3D network, trapping water inside (Figure 3).

Gelatin has similar advantages and disadvantages to collagen. It is biocompatible and biodegradable, as amino acids can also be found in humans, and are capable of degrading them into non-immunogenic byproducts. Their main advantages also include their reversible gelation properties, as, upon administration as injectable hydrogels, they melt at body temperature, leading to a temperature-triggered drug release. Gelatin is mainly used for bone regeneration and encapsulating small-molecule drugs, proteins, and nucleic acids, making it a versatile drug delivery system. Compared to collagen, it has poorer mechanical strength and faster degradation, which could lead to a potential burst release of incorporated drugs, thus, loss of structural integrity before the therapeutic effect is achieved. Batch-to-batch variability can also be a problem, with production of uniformly composed gelatin representing a challenge. If non-pharmaceutical-grade gelatin is applied, immunogenic reactions could occur due to impurities and residual animal proteins [57,58,59].

### 3.2. Recent Advancements in Gelatin-Based Hydrogels as Drug Delivery Systems

Generally, gelatin is utilized similarly to collagen, meaning the main indication areas include bone and cartilage regeneration, but other applications may also occur. Prolonging the drug or cell release from the hydrogel matrices can also be beneficial where it is required to have a sustained release profile, such as in the case of inflammatory bowel diseases. In the work of Treesuppharat et al., bacterial cellulose was combined with gelatin to form hydrogel composites. As a crosslinker, glutaraldehyde was utilized. The copolymerization was successfully executed, leading to uniformly sized and shaped composites. The hydrogen bonds between the amine and hydroxyl groups helped stabilize the composition. The bacterial cellulose helped improve gelatin’s swelling ability with a swelling ratio of up to 600%. This active substance-free drug delivery system provides adequate mechanical properties and is suitable for various administration routes [60]. Epichlorohydrin is a chemical crosslinker used in Bakravi et al.’s work to enhance the stability of gelatin-based hydrogels containing copper-oxide nanoparticles. With a high swelling ratio, the biodegradable hydrogel could be applied to a wound to exert the antimicrobial effect of copper oxide for both Gram-positive and Gram-negative bacteria. The elasticity and strength can prolong the residence time in the wound area, aiding wound healing and infection control [61]. Incorporating inorganic particles shows excellent potential in the formulation of gelatin hydrogels. Zeinali Kalkhoran et al. synthesized graphene-grafted gelatin nanocomposite hydrogels to enhance their potential as a drug delivery system. Due to the gelatin/graphene nanoplatelets, the swelling ratio was exerted to a high extent. The swelling of the hydrogel showed a swelled-state-dependent drug release profile. In the first hours, whilst swelling, an initial burst release was experienced, followed by a steady, sustained release after up to 24 h [58].

Ullah et al. exploited the advantages of gelatin-based hydrogels to deliver the anticancer agent oxaliplatin to the colon. As a crosslinker, N, N’-methylenebisacrylamide was used with the initiator ammonium persulfate. Successful entrapment of oxaliplatin was confirmed via Fourier transform infrared spectroscopy and thermal analysis. Acrylic acid modification was used on the hydrogels to enhance the swelling, drug loading, and release from the hydrogel matrix. An in vitro enzymatic degradation study showed higher resistance against collagenase and lysozyme, whilst the formulation is cytocompatible with a dose dependence of oxaliplatin. The preliminary safety evaluation showed an oral tolerability of up to 4000 mg/kg of body weight without causing hematological or histopathological adverse effects in rabbits [57]. Vocal fold scarring is a challenging therapeutic area that may be treated with basic fibroblast growth factor injection. However, the time of residence and action are limited, which indicates a technological need to implement hydrogels. In the work of Kobayashi et al., vibratory examination demonstrated a significant improvement of the vocal cords due to the prolonged effect acquired from the utilization of gelatin hydrogels [62]. Another possible indication includes the treatment of cardiac diseases, such as the stimulation of cardiac repair via erythropoietin. Kobayashi et al. demonstrated that applying an erythropoietin–gelatin hydrogel drug delivery system directly to the heart decreased myocardial infarct size. The left ventricular remodeling and function improved over the 2 weeks to 2 months after myocardial infarction. Applying the soft gel on the infarct site prolonged the effective therapy of the damaged muscle area [63]. Treatment of static nerve block also requires a prolonged drug delivery system to relieve postoperative pain. Local anesthetics alone have a limited duration of action, which gelatin hydrogels can control. Zhang et al. incorporated bupivacaine into gelatin hydrogels, which are characterized by a slow-release profile. The sustained release also decreased the toxicity of the local anesthetics. The porous three-dimensional mesh structure also helped with high drug loading capacity, further reducing the need for frequent administration. In vivo results demonstrated no obvious evidence of permanent inflammation or nerve damage in the block site’s sections [64].

Co-formulation with various polymers has also been proven helpful for gelatin-based hydrogels in increasing their stability and targetability. Changez et al. incorporated gentamicin sulphate in a poly(acrylic acid)-gelatin hydrogel to evaluate this system’s achievable release profile in vitro. As crosslinkers, N, N’-methylene bisacrylamide and glutaraldehyde were utilized to form an interpenetrating network. Based on the results, the release profile of gentamicin sulphate is characterized by diffusion controlled based on the Fickian diffusion mechanism. The increase in the gelatin percentage in the polymer resulted in a faster drug release profile, with 85% of the drug released within a week at physiological conditions [59]. Poly(N-isopropylacrylamide (PNIPAM) is also advised if the aim is controlled drug delivery, because it exerts a synergic effect with gelatin. Gheysoori et al. developed a thermoresponsive carrier based on this principle via photopolymerization. The hydrogel shows a reversible sol–gel transition close to body temperature. Hydrogels with a lower ratio of PNIPAM to gelatin also showed a more uniform and delayed release than other hydrogels, indicating their suitability for drug delivery purposes [65]. Sustained release delivery systems can also be achieved by adding hyaluronic acid, which was utilized by Zhang et al. to find a potential treatment for inflammatory bowel disease. The effect of hyaluronic acid/gelatin composites was investigated in their study to check the controllability of carboxymethyl chitosan microspheres. Based on the in vivo pharmacokinetic experiments, a sustained release of the model drug was found in the colon tissue for more than 24 h, playing an anti-inflammatory role based on the histopathological changes. Pharmacodynamic investigations also proved its efficiency in colitis in mice [66]. Gelatin hydrogels combined with other polymers are suitable for oral drug delivery and alternative administration routes, such as ocular delivery. Cheng et al. formulated chitosan–gelatin hydrogels containing curcumin and latanoprost to act as a dual-drug delivery system for glaucoma treatment. The developed system provided a sustained release on the ocular surface, a critical point in ocular delivery, as most of the administered drug is washed away via lacrimation. The formulation also decreased inflammation and apoptosis levels of cells, whilst showing in vivo biocompatibility with the eye [67]. A summary of the literature mentioned can be found in Table 3.

## 4. Albumin-Based Hydrogels for Medicinal Applications

### 4.1. General Structure, Gelation Mechanism, and Properties of Albumin Hydrogels

Serum albumin is an endogenous and non-glycosylated protein with a molecular weight of approximately 66.5 kDa. It is a globular, water-soluble protein that maintains osmotic pressure, transporting molecules, including drugs and pH buffer, in the plasma. Its structure is a single polypeptide chain with 585–609 amino acids, depending on which species produces it. Since it is amphiphilic, it can bind hydrophilic and hydrophobic compounds suitable for drug delivery. The most preferable type in therapy is human serum albumin, produced by human plasma, since it has a reduced immunogenic effect due to its human origin. Bovine serum albumin is purified from cow blood and closely related to human serum albumin, but people can be sensitive to it [68,69,70,71].

**Figure 4:**
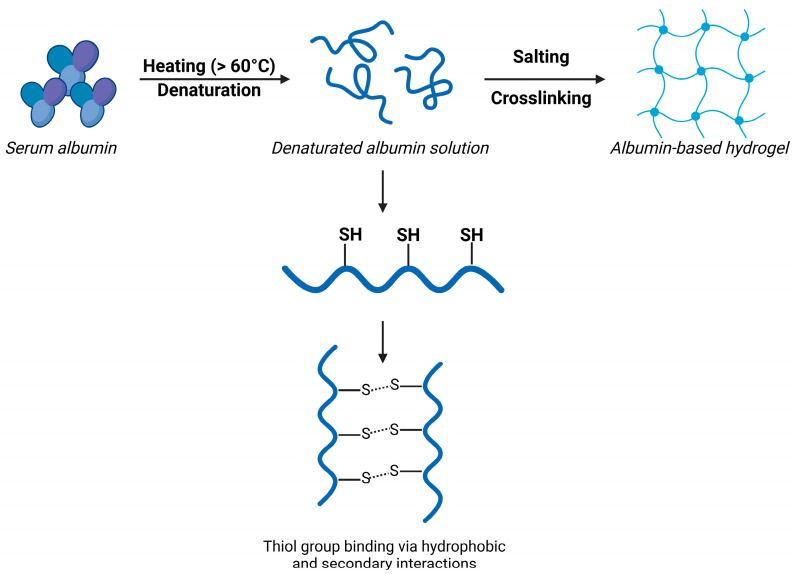
Gelation mechanisms of serum albumin. Created with biorender.com with permission.

There are three main mechanisms by which albumin can produce hydrogels. The first is the so-called thermal denaturation or heat-induced gelation. Above 60–70 °C, albumin undergoes denaturation, where the globular structure unfolds, leaving the hydrophobic residues and thiol groups exposed. These groups can be connected based on disulfide exchange reactions, hydrogen bonding, and hydrophobic interactions. After the interconnection of aggregates, a three-dimensional protein network forms, trapping water inside. As a result, elastic or brittle gels can be formed, which are irreversible (Figure 4). pH-induced or isoionic gelation occurs on the isoelectric point (4.7) of albumin, where it has no net charge, which reduces repulsion. This leads to aggregation and a similar three-dimensional network formation, especially at higher ionic strength. Crosslinking is the third method to form hydrogels, which can be prepared via enzymatic crosslinking, such as with transglutaminase, or with chemical crosslinkers, such as glutaraldehyde, genipin, or EDC. As a result, stable, tunable hydrogels can be produced and are suitable for drug administration [71,72,73,74].

As mentioned before, human serum albumin is biodegradable and biocompatible due to its origin. Thus, its utilization is favorable. It is also injectable and has the capacity to form crosslinked hydrogels or nanoparticles, enhancing permeability. The formulated hydrogels are transparent and easily characterized by optical and rheological properties. However, some challenges arise from their utilization, such as batch variability, the cost of production, and the limited thermal stability, which is utilized in thermal denaturation, but can also cause degradation if the gels are not appropriately handled. Albumin-based gels are also soft gels; thus, they require modifications to ensure sustained drug release or structural integrity [72,74,75].

### 4.2. Application of Albumin-Based Hydrogels for Therapy

One of the main applications of albumin-based hydrogels is drug delivery, which is most likely to be utilized in cancer therapy. As sustained release systems, they can be engineered to maintain a high drug concentration around the tumor cells, decreasing the need for frequent administration and chemotherapy-related adverse effects. In the study by Qian et al., paclitaxel-loaded red blood cell membrane nanoparticles were hybridized with albumin hydrogel to provide an efficient treatment option for gastric cancer with peritoneal metastasis. The gelation occurred 12 min after the subcutaneous injection of the carrier system. It showed an extended, sustained release profile, where the released paclitaxel was only approximately 30% after 6 days. In vivo antitumor evaluation was also performed with a subcutaneous xenograft and peritoneal dissemination model, showing that the hydrogel suppresses tumor growth after a single injection [76]. Yu et al. investigated albumin hydrogels for the treatment of prostate cancer, where the injected hydrogel remained stable six weeks post-injection. Moderately hypofractionated radiotherapy was applied while using the hydrogel, which showed accelerated degradation followed by remaining in the body for three more months [77].

Another critical application is tissue regeneration, as albumin-based hydrogels have gained much interest in this area in recent years. They offer a heterozygous-free, safe, biocompatible, and biodegradable platform to aid tissue regeneration whilst encapsulating various cells or active substances to trigger and enhance regeneration [78]. Zhang et al. manufactured injectable albumin-based hydrogels with a characteristic and rapid self-healing capability, demonstrating the ability to completely repair their structure within 2 min with up to 100% self-healing efficiency. The formulation is cytocompatible, with minimal cytotoxic effects. The administered hydrogel possesses a dual function. First, it provides mechanical resilience through self-healing and creates a biologically compatible environment, promoting wound regeneration [79]. In the work of Kenawy et al., albumin-based hydrogels constructed a hydrogel composite based on bovine serum albumin, promoting wound healing and aiding the vascular endothelium [80]. Yoon et al. proved significant promotion for the growth of blood vessels, as it acted as a responsible factor for host cell infiltration after implementation, ensuring the highest efficacy for tissue regeneration [75].

Deng et al. demonstrated the efficient utilization of injectable, redox albumin-based hydrogels loaded with dihydromyricetin, where they used glutathione to reduce albumin molecules further to prolong the release profile of the encapsulated active substance. The encapsulation of dihydromyricetin in the bovine serum albumin hydrogel significantly enhanced its bioavailability compared to the native solution of the drug. It also stimulated the inhibition of bacteria, including *E. coli* and *S. aureus,* to provide an effective treatment against bacterial infections [81]. A summary of the data from the literature is found in Table 4.

## 5. Silk Fibroin-Based Hydrogels for Medicinal Applications

### 5.1. Structure, Gelation Mechanism, and Properties of Silk Fibroin

Silk fibroin is a polypeptide containing heavy (H-chain, 390 kDa) and light (L-chain, 26 kDa) chains alternating in a ratio of 1:1, where the L-chain is linked to the C-terminus of the H-chain via a single disulfide bond [82]. It is produced by *Bombyx mori* silkworms, which are the leading producers of it [83,84,85]. Via the degumming of raw silk, meaning removing sericin, the obtained silk fibroin is a non-toxic, thermally stable, biocompatible, biodegradable protein which also has attractive features in its mechanical strength and toughness [86,87].

**Figure 5:**
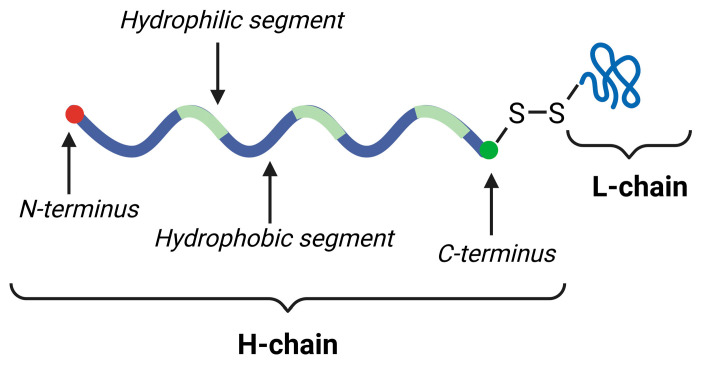
The general structure of silk fibroin originated from *Bombyx mori* silkworms. Created with biorender.com with permission.

Numerous mechanisms can be used to form hydrogels of silk fibroins, including physical and chemical methods. Physical crosslinking can be performed via temperature changes, a key factor in all silk fibroin-based hydrogel formation. The increase in temperature promotes the frequency of effective molecular collisions, further enhancing the aggregation of silk fibroin molecules. On the other hand, the hydrophobic segment of the silk fibroin also increases with temperature, strengthening the hydrophobic interactions [21,88,89]. Silk fibroin also exhibits excellent sensitivity to outer mechanical forces, because native fibroin solution is characterized by only subtle tensile and shear flow forces to prevent premature gelation. After applying high shear forces, this mechanism is hindered from inducing the conformation transition of fibroin into disordered helical structures, changing into a β-sheet structure to form hydrogels [90,91]. Chemical crosslinking can also be exploited in two ways. Enzymatic crosslinking, using horseradish peroxidase, glutaminase, or tyrosinase, has also attracted considerable interest in biomedical applications due to the high stability of the hydrogels ensuring controlled release [86,92,93]. Other non-enzymatic chemical crosslinkers include genipin, glutaraldehyde, and EDC, which act similarly to other protein-based delivery systems, ensuring proper stability and controlled release from the hydrogel matrices [87,94,95,96]. Silk fibroin has a tunable gelation and degradation process, which can be influenced by pH, the shear force applied, and sonication during production. It can also be chemically modified to ensure higher stability and a slow and sustained drug release profile. It is biocompatible and biodegradable, also characterized as non-immunogenic [89]. There are also several disadvantages, such as batch variability and low initial cell encapsulation efficiency without additives. The purification process is long, time-consuming, and costly, especially at higher desired yields [87]. The general structure of silk fibroin can be found in Figure 5.

### 5.2. Application of Silk Fibroin-Based Hydrogels in Medicine

Similar to those based on collagen, silk fibroin-based hydrogels can also be utilized for bone regeneration. With proper engineering, silk fibroin can be beneficial due to its high toughness and mechanical strength, making silk fibroin hydrogel-derived bone tissue engineering scaffolds a popular choice [97,98,99,100]. In the work of Jiang et al., silk fibroin was co-formulated with gelatin to form a composite matrix suitable for the proliferation and differentiation of bone marrow mesenchymal stem cells. Three months post-implantation into the skull defect, the hydrogel improved the bone regeneration quality with less tissue response [101]. Zhang et al. developed an in situ silk hydrogel that encapsulated bone morphogenetic protein-2 and vascular endothelial growth factor to promote bone formation and angiogenesis after traumatic events. Their results showed a promising treatment option for irregular bone defects due to the adequate mechanical properties of the in situ gels [102]. In the work of Kim et al., γ-ray irradiation was used to manufacture hydroxyapatite nanoparticles, which were embedded in silk fibroin hydrogels, also for bone regeneration. Their results proved that the nanoparticle composite hydrogel promoted osteogenic differentiation compared with pure silk fibroin hydrogels [103]. Meinel et al. also proved that porous silk fibroin-based scaffolds alone can induce bone formation within 5 weeks in defects in a mouse skull [104].

Another important indication is the regeneration of cartilage based on similar principles of bone regeneration, but, in this case, the proliferation and differentiation of chondrocytes gain more attention. Studies have also demonstrated that silk fibroin hydrogels offer identical functional properties to the commonly applied agarose-based engineered cartilage [105]. In the work of both Shen et al. and Zhang et al., the multifunctional hydrogels enhanced osteochondral regeneration and bone marrow mesenchymal stem cell-specific affinity [99,106]. Three-dimensional printing has emerged as one of the novel production methods for various applications in medicine, including tissue and bone regeneration [107,108]. Lee et al. printed intact ear cartilage using polyvinyl alcohol and silk fibroin-composed hydrogels [109]. Similarly, Hong et al. produced glycidyl methacrylate-modified silk fibroin hydrogels to promote the encapsulated cells’ viability, proliferation, and differentiation in the cartilage for up to 4 weeks [108].

Nerve regeneration is also of interest in the utilization of silk fibroin-based hydrogels. Peripheral nerve injuries require advanced treatment, including autologous transplantation, whilst central nervous system-associated nerve injuries are complicated due to various endogenous factors inhibiting repair. Zhang et al. developed a novel silk fibroin nerve conduit that enables targeted delivery of nerve growth factors to promote efficient nerve repair [110]. Zhou et al. similarly synthesized fibroblast growth factor-loaded methacrylate-modified silk fibroin hydrogels, also capable of promoting neurite regeneration and inhibiting glial cell proliferation to improve the neuronal mitochondrial function [111]. Tang et al. formulated a silk-based light-triggered gelling system capable of achieving enhanced cell recruitment and myelination via its anisotropic topography and adhesion ligands. This proves the importance of topological microstructure design, one of the main focus areas of nerve regeneration scaffolds [112]. Spinal cord regeneration is also one of the most promising applications of silk fibroin, which was proved by the works of Gao et al. [113]. A summary of the results presented can be found in Table 5.

## 6. Fibrin-Based Hydrogels for Medicinal Applications

**Figure 6:**
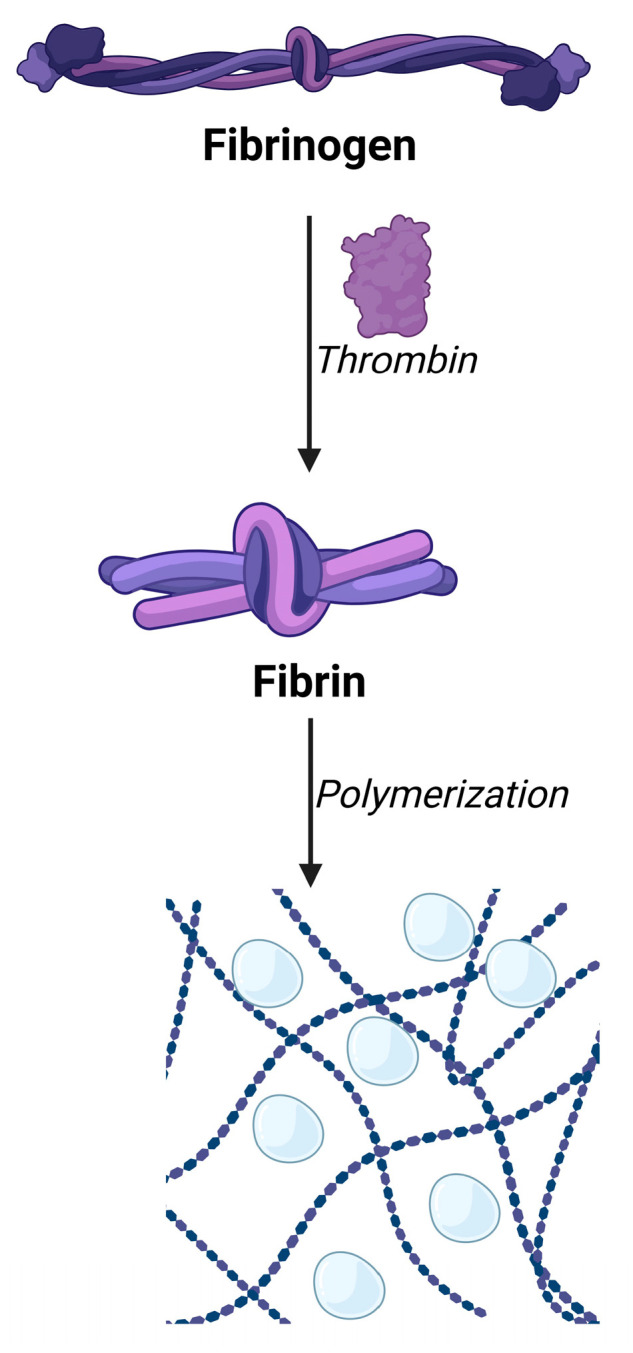
Schematic mechanism of fibrinogen → fibrin transformation followed by polymerization into a three-dimensional hydrogel structure. Created with biorender.com with permission.

Fibrin plays a central role in blood clotting, and a similar mechanism is crucial in wound healing, cell/drug delivery, and tissue engineering. Fibrin is an insoluble fibrous protein produced by fibrinogen, one of the soluble plasma glycoproteins. Commercially, to achieve pharmaceutical-grade fibrin, thrombin and fibrinogen are acquired, which react in the absence of calcium ions to produce pure fibrin capable of gel formation (Figure 6). Since it is also biocompatible and biodegradable, its application is preferable, also promoting cell adhesion and proliferation due to its cell-binding domains, facilitating attachment, proliferation, and migration. Generally, the formulations are relatively soft and fragile, and the main problem is the fast degradation rate in the human body. Xenogeneically sourced fibrin can also be problematic as it would cause immunogenic reactions [114,115,116,117].

### Recent Advancements in Fibrin-Based Gel Systems for Medical Applications

Macrophage accumulation plays a key role in regulating tissue regeneration and inflammation; thus, fibrin hydrogel scaffolding can be used to promote this activity in order to reduce the inflamed area and aid regeneration. The formulation of Tanaka et al. decreased the secretion of tumor necrosis factor-α, which is one of the pro-inflammatory cytokines, while increasing the secretion of interleukin-10, an anti-inflammatory cytokine. Through successful administration of fibrin hydrogels, macrophage infiltration expressed rapidly to a high extent, indicating that the fibrin hydrogels have a strong promoting effect on the recruitment of macrophages, most specifically those with anti-inflammatory effects [118]. Combined with chitosan, fibrin hydrogels can also benefit human dental pulp regeneration, as they promote neoformation of dental pulp tissue. In Ducret et al.’s work, this theory was proven to positively affect dental pulp cell morphology, proliferation, viability, and collagenous matrix production [114].

Fibrin-based hydrogels can also be helpful for myocardial infarction treatment. In contrast, vascular endothelial growth factor and cardiomyocytes can be embedded in the hydrogel to promote the proliferation of healthy myocardial cells. This was the basis for the work of Liu et al., who combined the beneficial effects of fibrin with sodium alginate to form a mechanically stable gel. The injected gel induced angiogenesis whilst supporting the retention and integration of the transplanted cardiomyocytes into the host myocardium. The increased blood recovery helped mitigate the ischemic microenvironment, thus improving the viability of transplanted cells [119]. Applications to the central nervous system are also critical, as nerve damage such as spinal cord injury is still a challenging therapeutic area in current medicine. In the work of Sudhadevi et al., neural progenitor cells were embedded in fibrin hydrogels to reduce the post-transplant immune response after spinal cord injury. As a result, degradable, porous but robust fibrin strands were developed, helping neural cell attachment, migration, and, last but not least, tissue regeneration. It was also concluded that the addition of excess thrombin resulted in fibrinogen clotting with a reduced pore size and pore density, significantly affecting cell survival. The proper formulation regulated the immune response to further enhance the efficacy of neural regeneration [120]. In the work of Nazari et al., a similar approach was utilized where the hydrogel-embedded basic fibroblast growth factor, epidermal growth factor, and platelet-derived growth factor simulated the differentiation of induced pluripotent stem cells into oligodendrocytes, aiding in the case of neural tissue regeneration [115]. A summary of the applications of fibrin can be found in Table 6.

## 7. Elastin-Based Hydrogels for Medicinal Applications

### 7.1. General Structure, Gelation Mechanism, and Properties

Elastin is also an animal-derived protein capable of forming a hydrogel in the connective tissues of the mammal body, especially in organs or areas where severe stretching and recoil occur, such as the skin, arteries, or lungs. Due to its anatomical and physiological functions, it is highly elastic (as its name suggests) and has high fiber content. Elastin is rich in amino acids, especially hydrophobic ones, including valine, alanine, proline, and glycine, and its precursor, tropoealastin, is secreted into the extracellular matrix, which is enzymatically crosslinked via lysyl oxidase, forming mature elastin. To form rubber-like elasticity and durability, unique amino acids, such as desmosine and isodesmosine, are involved [121,122].

The hydrogel formation of elastin starts with the dissolution of tropoelastin, found in a monomeric state, existing in disordered coils where the hydrophobic and hydrophilic regions are distributed evenly along the chain structure. Because these are temperature-dependent, self-assembling systems, coacervation is applied as a primary formulation technique, where the hydrophobic segments of elastin aggregate into micellar-like aggregates, forming a dense, condensed network. It is still not yet stable; thus, crosslinking must be implemented. In the case of native elastin, lysyl oxidase can be utilized, just like in mammal bodies. However, other chemical crosslinking is also acceptable, such as crosslinking with glutaraldehyde, genipin, or other carbodiimides [87,123,124].

**Figure 7:**
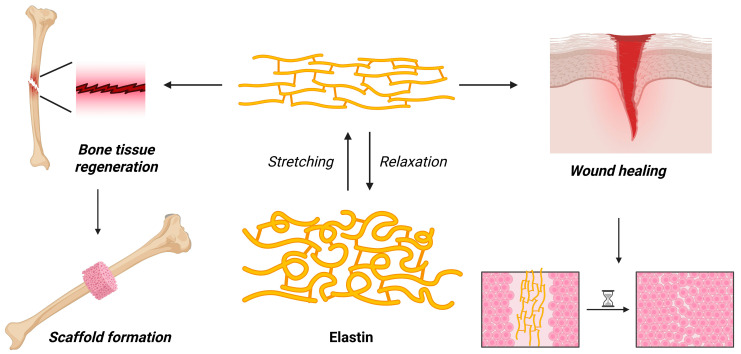
Elasticity of elastin and its main potential applications in medicine. Created with biorender.com with permission.

Elastin-based hydrogels offer excellent elasticity and resilience, and can mimic the behavior of native tissues, such as the organs of their origin, like lungs and blood vessels. They are also biocompatible and biodegradable, non-immunogenic proteins. Their main advantage lies in their tunability, including the fact that they can form innovative carrier systems, which can be tuned to react to temperature changes in the biological fluids, mediated at different pH, ionic strength conditions, or enzymatic sensitivity. Some limitations can be found in elastin, such as the limited mechanical strength in the same forms and the lack of rigidity, which can be solved via crosslinking. Crosslinking can also be a slow process, especially in the case of natural elastin, and the advantage of thermal sensitivity can also be a disadvantage, especially when formulating complex delivery systems [121,125,126]. A schematic visualization of elastin and its application is found in Figure 7.

### 7.2. Recent Advancements in the Application of Elastin-Based Hydrogels for Medical Applications

One essential utilization of elastin-like hydrogels is in the case of angiogenesis, which is a crucial factor in wound healing. Tian et al. produced acryloyl-(polyethylene glycol)-N-hydroxysuccinimide ester modified elastin hydrogels combined with methacrylated gelatin, which can mimic the dermal microenvironment. Proper mechanical strength and tunability were found regarding their formulation, with a modulus in the range of human skin. Mice wound model studies showed that the hydrogel attracted abundant neutrophils and predominant macrophages to promote infiltration into the hydrogel matrices. The application of the formulation also resulted in high rates of collagen deposition and dermal regeneration, thus making it applicable for medical use in wound therapy [127]. In the work of Stojic et al., similar results were found where elastin was combined with plasma to further enhance the skin tissue engineering properties of the formulation. To strengthen the elasticity of the hydrogel, azide and cyclooctyne modifications were implemented, decreasing gelation time and concentration by the increased mechanical strength. With the application of the hydrogel formulation, the proliferation of human primary keratinocytes increased significantly, thus making it suitable for wound healing or other dermocosmetic applications [128]. The combination of polymers with elastin, such as the utilization of hyaluronic acid, is also promising. Fiorica et al. developed a hyaluronic acid-modified formulation of elastin hydrogels capable of producing scaffolds, supporting adhesion and growth of human vascular endothelial cells due to its ability to control the diffusion rate of the incorporated vascular endothelial growth factor. Differences in various molecular weight hyaluronic acids are well-known, and the authors used low molecular weight hyaluronic acid to achieve a soft gel-like structure, compared to higher molecular weight polymers, which can result in hard gels unsuitable for this application [123].

Angiogenesis is also of paramount importance regarding critical limb ischemia, which is characterized by the impairment of microcirculation alongside the inflammation of muscular tissue and necrosis. Marsico et al. found a solution to this problem with an engineered hydrogel formulation based on elastin. An induction in arteriole formation, reduction of fibrosis, and anti-inflammatory macrophage polarization was experienced, suggesting the mediation of tissue repair. Their results elucidate the angiogenic potential of elastin-like hydrogels, especially focusing on the glycosylation alterations, forming possible new therapeutic indications and targets [129]. Other applications may include other ischemic diseases, such as ischemic heart disease, for which Contessotto et al. proposed a novel formulation based on elastin-like recombinamers. The formulated hydrogels were injectable in intramyocardial injections and investigated in a sheep’s non-transmural myocardial infarction model. Three weeks post-injection, less fibrosis and more angiogenesis were experienced in the core region of the ischemic area, and a complete functional recovery of ejection fraction was also observed. The hydrogel formulation also aided the preservation of cardiomyocytes in the border zone of the infarct [130]. In combination with collagen, elastin-like polypeptide hydrogels can be engineered, which may be helpful in bone tissue regeneration, exploiting the structural behavior of collagen with the advantages of elasticity originating from elastin. In the work of Pal et al., doxycycline and recombinant human bone morphogenetic protein-2 were loaded inside such a system, showing an interconnected, microporous architecture supporting stem cell attachment. As a result, the expression of osteogenic markers, such as alkaline phosphatase and osteocalcin, significantly increased, promoting bone tissue regeneration [131]. Another indication of elastin-like hydrogels could be malignant glioblastoma, an aggressive brain tumor that has a high resistance to chemotherapy, thus posing an unmet clinical need. Dragojevic et al. encapsulated doxorubicin inside the hydrogel matrix, showing adequately high swelling and mechanical properties leading to a sustained release profile of the encapsulated drug. The administered hydrogel significantly reduced glioblastoma cells’ survival rate and proliferation in vitro [124]. Results are summarized in Table 7.

## 8. Conclusions and Future Perspectives

In conclusion, numerous examples demonstrate the potential of protein-based hydrogels for medical use, which are generally biocompatible and biodegradable. The variety in composition, the ability to co-formulate them with other non-protein-based polymers, and the tunability to enhance or sustain drug release have been shown in the case of these carriers. Not only drug or cell delivery, but also regenerative medicine plays a crucial role in the field of protein-based hydrogels. Especially in the case of bone tissue, cartilage, myocardial, skin tissue, wound, or nerve engineering, promising technological advancements can be found in the current research and development landscape. Despite the advancements and the enhanced therapeutic options, the main burden still lies in regulating these novel technologies, which bounce between the fields of biotechnology, nanotechnology, and implantation medicine. Classification of these products is complex, since they do not contribute to medicine via classic pharmacological routes, and thus, they cannot be called drugs. Medical device classification suits them most of the time. Still, cells or drugs can be embedded into them, making them a combined product category, leading also to a more complex authorization process by regulatory agents (Food and Drug Administration, European Medicines Agency, etc.). There are also some ethical or religious obstacles to many animal-derived proteins that may require synthetic proteins or peptides to fulfill the patient’s needs in these areas. The future is heading in a good direction, where many unmet clinical needs, for which a drug cannot be easily administered, may find resolution. The application of hydrogels in traumatic events, such as bone fractures, cartilage detachment, spinal cord injury, or myocardial infarction, has also been proven to be feasible. With the emergence of biotechnology, recombinant technology also offers promising techniques to enhance the physiochemical and mechanical properties of protein-based gels.

## Figures and Tables

**Table 1:** General advantages and disadvantages of protein-based hydrogels for drug/cell delivery and regenerative medicine.

Advantages	Disadvantages (Challenge)
Biocompatibility and biodegradability	Susceptibility to enzymatic degradation in vivo
Supports cell adhesion and proliferation	Alone, they usually provide poor mechanical strength, requiring crosslinking
Engineered to control drug release (sustained generally)	Batch-to-batch variability due to their natural origin
Injectable forms are available	Can be unstable against thermal or pH changes
Tunable physicochemical properties ensuring targetability	Limited shelf life due to biological instability
Can be modified to behave in a stimuli-responsive manner (pH, temperature, or enzymes)	Extraction and purification processes are difficult and complex
Excellent water-holding and swelling capacity mediates high drug loading capability	Difficult to sterilize them, increasing the risk of contamination with pathogens if they are derived from animal sources
Provides an extracellular matrix-like environment for cells	Without stabilization, they can degrade too easily, leading to potential burst drug release
Often non-toxic and non-immunogenic	The high water content can reduce loading capacity for hydrophobic drugs
Sustainable sourcing is possible with environmentally friendly production methods	If the mechanical strength is increased too much, the slow diffusion of large molecules may be hindered
Can be combined with polymers to enhance their physicochemical and applicability parameters further	Severe regulatory challenges due to their biological origin

**Table 2:** Summary of examples of the utilization of collagen for drug/cell delivery, specifically for bone regeneration or wound healing.

Indication	Additional Agents	Main Result	Reference
Bone tissue regeneration	Sodium alginate	Enhanced osteogenic differentiation Increased bone volume by almost double	[32]
Bone tissue regeneration	Hyaluronic acid, chitosan	Significant increase in the proliferation of new bone material	[45]
Bone tissue regeneration	Hyaluronic acid-tyrosine	Excellent soft tissue filling without cytotoxic effect, without inflammatory response	[46]
Bone tissue regeneration	Sodium alginate, Hydroxyapatite	Enhanced bone regeneration and host–implant regeneration	[47]
Bone tissue regeneration	Chitosan, lysine, hyaluronic acid	Enhanced proliferation and adhesion of osteoblast-like cells and alkaline phosphatase expression	[51]
Lung carcinoma, breast tumor	Sodium alginate, immune stimulators	Prevented lung metastasis Eliminated the original breast tumor cells	[42]
Wound repair	-	High cell viability upon administration Enhanced wound healing properties	[43]
Wound repair	Guar gum, poly(N-isopropylacrylamide), graphene oxide, borax	Reversible or permanent network formation was detected based on the excipient Self-healing hydrogels exerted a higher healing ratio	[52]
Cartilage regeneration	Activated chondroitin sulphate, chondrocytes	Increased extracellular matrix secretion of chondrocytes	[44]
Cartilage regeneration	Hyaluronic acid	Enhanced cartilaginous tissue formation Effective connection formation amongst newborn cell clusters	[48]
Cartilage regeneration	Carbon dot nanoparticles	Effective addition to photodynamic therapy to promote cartilaginous tissue formation	[49]
Cartilage regeneration	Icariin, hyaluronic acid	Enhanced cartilage formation and osteoblast proliferation	[50]

**Table 3:** Summary of various examples demonstrating the applicability of gelatin hydrogels for drug delivery.

Indication	Additional Agents	Main Result	Reference
-	Bacterial cellulose	Increased swelling due to the combination of collagen and cellulose	[60]
Wound healing	Cooper-oxide nanoparticles	Increased wound healing and infection control properties due to sufficient antimicrobial effect on Gram-positive and harmful bacteria	[61]
-	Graphene	Controllable drug release profile (initial whilst swelling—burst-type, followed by sustained release)	[58]
Cancer	Oxaliplatin, acrylic acid	Enhanced swelling, drug loading, and release due to the modification of acrylic acid High resistance against degrading enzymes (collagenase, lysozyme)	[57]
Vocal fold scarring	Basic fibroblast growth factor	Significant improvement of the vocal cords for a prolonged time after injection	[62]
Post-myocardial infarct regeneration	Erythropoietin	Improved left ventricular remodeling and function 2 months post-infection	[63]
Static nerve block	Bupivacaine	Decreased toxicity of the local anesthetic Sustained release of anesthetic drug	[64]
Infection control	Gentamicin sulphate	Sustained release of the antibiotic agent, providing longer treatment	[59]
-	(*model active substance*), poly(N-isopropylacrylamide)	Sustained and uniform drug release profile with the polymer-enriched gelatin hydrogel	[65]
Inflammatory bowel disease	(*model active substance*), hyaluronic acid	Colon-targeted, sustained drug release profile was achieved with the hyaluronic acid modified gelatin hydrogel	[66]
Glaucoma	chitosan, curcumin, latanoprost	Decreased washing away via lacrimation Decreased inflammation and apoptosis after administration	[67]

**Table 4:** Summary of various examples demonstrating the applicability of serum albumin-based hydrogels for medical utilization.

Indication	Additional Agents	Main Results	Reference
gastric cancer with peritoneal metastasis	Paclitaxel embedded in red blood cell membrane nanoparticles	Rapid gelation after subcutaneous injection Prolonged therapeutic efficacy, suppressing tumor growth after a single injection	[76]
prostate cancer	-	Stable hydrogel utilization at the site of radiotherapy	[77]
tissue regeneration	-	Rapid self-healing capability of injectable hydrogels Promoted wound healing due to enhanced microenvironment	[79]
wound healing	-	Promoted wound healing Enhanced vascular endothelium repair	[80]
tissue regeneration	-	Promoted growth of blood vessels	[75]
bacterial infections	dihydromyricetin	Sustained drug release profile from hydrogel matrices Stimulated inhibition of *E. coli* and *S. aureus*	[81]

**Table 5:** Summary of various examples demonstrating silk fibroin-based hydrogels for medical applications.

Indication	Additional Agents	Main Results	Reference
bone regeneration	gelatin	Enhanced proliferation and differentiation of bone marrow mesenchymal cells	[101]
bone regeneration	bone morphogenetic protein-2 and vascular endothelial growth factor	Promoted cell proliferation and differentiation after traumatic events alongside enhanced angiogenesis	[102]
bone regeneration	hydroxyapatite nanoparticles	Promoted osteogenic differentiation with the hydroxyapatite nanoparticles	[103]
bone regeneration	-	Induced bone formation within 5 weeks in mouse skull defects	[104]
cartilage regeneration	-	Enhanced osteochondral regeneration and bone marrow mesenchymal stem cell-specific affinity	[99,106]
cartilage regeneration	polyvinyl alcohol	3D printed intact ear cartilage using silk fibroin	[109]
peripheral nerve injury	-	Targeted delivery of nerve growth factors, promoting efficient nerve repair	[110]
spinal cord regeneration	fibroblast growth factor	Promoted neurite regeneration and inhibited glial cell proliferation, improved the neuronal mitochondrial function	[111]
nerve regeneration	-	Enhanced cell recruitment and myelination	[112]

**Table 6:** Summary of various examples regarding the utilization of fibrin hydrogels for medical applications.

Indication	Additional Agents	Main Results	Reference
inflammation	-	Decreased secretion of tumor necrosis factor-α and other pro-inflammatory cytokines	[118]
dental pulp regeneration	chitosan	Better pulp cell morphology, enhanced proliferation and viability Enhanced collagenous matrix production	[114]
myocardial infarction	vascular endothelial growth factor, cardiomyocytes	Promoted proliferation of healthy myocardial cells Supported retention and integration of transplanted cardiomyocytes Increased blood recovery mitigated the ischemic microenvironment	[119]
spinal cord injury	neural progenitor cells	Reduced post-transplant immune response after spinal cord injury	[120]
nerve tissue regeneration	basic fibroblast growth factor, epidermal growth factor, platelet-derived growth factor	Enhanced stimulation of the differentiation of induced pluripotent stem cells and oligodendrocytes	[115]

**Table 7:** Summary of various examples regarding the utilization of elastin-based hydrogels for medical applications.

Indication	Additional Agents	Main Results	Reference
wound healing	acryloyl-(polyethylene glycol)-N-hydroxysuccinimide ester	Enhanced wound regeneration via the attraction of abundant neutrophils and predominant macrophages	[127]
skin tissue regeneration	human plasma	Enhanced proliferation of human primary keratinocytes	[128]
vascular regeneration	hyaluronic acid	Enhanced adhesion and growth of human vascular endothelial cells	[123]
limb ischemia	-	Reduction of fibrosis and anti-inflammatory macrophage polarization	[129]
ischemic heart disease	-	Decreased degree of fibrosis Complete functional recovery of ejection fraction Preservation of cardiomyocytes	[130]
bone tissue regeneration	Recombinant human bone morphogenetic protein-2	Significantly increased expression of osteogenic markers	[131]
malignant glioblastoma	doxorubicin	Sustained release profile of the encapsulated drug Significant reduction of survival rate and proliferation of glioblastoma cells	[124]

## Data Availability

Data are available upon request from the corresponding author.

## Abbreviations

The following abbreviations are used in this manuscript:

3D	3-dimensional
4T1	stage IV human breast cancer cell line
ALP	alkaline phosphatase
BDEE	1,4-butanedioldiglycidyl ether
CT-26	undifferentiated colon carcinoma cell line
DNA	deoxyribonucleic acid
*E. coli*	*Escherichia coli*
EDC	1-ethyl-3-(3-dimethylaminopropyl)carbodiimide
HLC	human-like collagen
kDa	kilodalton
PNIPAM	poly(N-isopropylacrylamide)
RNA	ribonucleic acid
ROS	reactive oxygen species
*S. aureus*	*Staphylococcus aureus*
